# Post-mortem enamel surface texture alteration during taphonomic processes—do experimental approaches reflect natural phenomena?

**DOI:** 10.7717/peerj.12635

**Published:** 2022-01-14

**Authors:** Katrin Weber, Daniela E. Winkler, Ellen Schulz-Kornas, Thomas M. Kaiser, Thomas Tütken

**Affiliations:** 1 Applied and Analytical Palaeontology, Institute of Geosciences, Johannes Gutenberg University, Mainz, Germany; 2 Center of Natural History (CeNak), University of Hamburg, Hamburg, Germany; 3 Department of Natural Environmental Studies, Graduate School of Frontier Sciences, The University of Tokyo, Tokyo, Japan; 4 Department of Cariology, Endodontology and Periodontology, University of Leipzig, Leipzig, Germany; 5 Department of Human Evolution, Max Planck Institute for Evolutionary Anthropology, Leipzig, Germany

**Keywords:** Dental microwear, Diet, Post-mortem wear, Vertebrate enamel, Experimental alteration, Fluvial transport, Aeolian sediment transport, Chemical dissolution

## Abstract

Experimental approaches are often used to better understand the mechanisms behind and consequences of post-mortem alteration on proxies for diet reconstruction. Dental microwear texture analysis (DMTA) is such a dietary proxy, using dental wear features in extant and extinct taxa to reconstruct feeding behaviour and mechanical food properties. In fossil specimens especially, DMTA can be biased by post-mortem alteration caused by mechanical or chemical alteration of the enamel surface. Here we performed three different dental surface alteration experiments to assess the effect of common taphonomic processes by simplifying them: (1) tumbling in sediment suspension to simulate fluvial transport, (2) sandblasting to simulate mechanical erosion due to aeolian sediment transport, (3) acid etching to simulate chemical dissolution by stomach acid. For tumbling (1) we found alteration to be mainly dependent on sediment grain size fraction and that on specimens tumbled with sand fractions mainly post-mortem scratches formed on the dental surface, while specimens tumbled with a fine-gravel fraction showed post-mortem formed dales. Sandblasting (2) with loess caused only negligible alteration, however blasting with fine sand quartz particles resulted in significant destruction of enamel surfaces and formation of large post-mortem dales. Acid etching (3) using diluted hydrochloric acid solutions in concentrations similar to that of predator stomachs led to a complete etching of the whole dental surface, which did not resemble those of teeth recovered from owl pellets. The experiments resulted in post-mortem alteration comparable, but not identical to naturally occurring post-mortem alteration features. Nevertheless, this study serves as a first assessment and step towards further, more refined taphonomic experiments evaluating post-mortem alteration of dental microwear texture (DMT).

## Introduction

Dental microwear texture analysis (DMTA) is a common and well-established proxy for reconstructing diet and food properties in extant and extinct vertebrates (Scott et al., 2005; Merceron et al., 2007; Ungar et al., 2003; Ungar et al., 2020; Ungar, Merceron & Scott, 2007; Ungar, Grine & Teaford, 2008; Scott et al., 2009; Schulz, Calandra & Kaiser, 2010; Winkler et al., 2013; Winkler et al., 2016). It has been successfully applied to terrestrial large and small mammals (Merceron & Madelaine, 2006; Calandra et al., 2012; Purnell et al., 2013; Kubo et al., 2017; Schulz-Kornas et al., 2019), including (semi-) aquatic mammals (Purnell et al., 2017; Bethune et al., 2019), but also non-mammalian taxa like lepidosaurs (Bestwick, Unwin & Purnell, 2019; Winkler et al., 2019), mammal-like reptiles (Kalthoff et al., 2019), and fish (Purnell & Darras, 2016). Microwear and microwear texture are shown to be powerful proxies for oral behaviour and ingesta-intake in general; however, analyses can be biased by post-mortem surface alteration resulting in enamel loss (King, Andrews & Boz, 1999; Dauphin et al., 2018). The resulting taphonomic features can be visually identified by trained operators, and as such taphonomically altered specimens removed prior to analysis. In fossil sample sets, it is often the case that a high number of specimens have to be excluded from analyses, since they show potential post-mortem alteration, which reduces the sample size drastically (Ungar, Grine & Teaford, 2008). Chemical or mechanical alteration can be caused by transport, burial and/or different fossilisation processes (*e.g.*, Denys, 2002; Dauphin et al., 2018), and the impact of such post-mortem alteration has not been sufficiently explored. Only a few studies have analysed post-mortem alteration under natural conditions (Dauphin et al., 2003; Dauphin et al., 2018; Martínez & Pérez-Pérez, 2004) or in different experimental approaches (*e.g.*, Gordon, 1983; Gordon, 1984; King, Andrews & Boz, 1999; Böhm et al., 2019; Kropacheva et al., 2019; Uzunidis et al., 2021). However, it is unknown how well natural alteration processes can be experimentally simulated, as artificial post-mortem alteration can hardly recreate processes that act on geological timescales. Thus, it needs to be tested how comparable simplified experimentally induced post-mortem wear alteration is to naturally induced alteration. Here we performed three dental microwear texture (DMT) alteration experiments to approach simplified recreation of three different mechanical and chemical natural taphonomic processes:

1. Fluvial transport in a sediment suspension (by tumbling).
2. Aeolian erosion by windblown mineral dust (by sandblasting).
3. Stomach digestion erosion in an acidic environment (by acid etching).

Fluvial transport (1) of vertebrate fossil remains is a very common post-mortem alteration process in terrestrial depositional settings. It can occur in permanent as well as ephemeral river systems, *e.g.*, in arid settings with heavy rainfall or intense snowmelt events (Milner, 1987; Astin et al., 2010; Cressler et al., 2010; Bennett et al., 2016; Beylich & Gintz, 2016). In high-energy environments and during long fluvial transport processes, water, together with transported sediment, has a strong abrasion effect on objects, *e.g.*, as seen in its rounding effect on sand, stones or bones (Domokos et al., 2014). This type of mechanical post-mortem alteration process modifies skeletal remains vertebrates transported in fluvial systems as well as reworked fossil specimens. To experimentally simulate sediment contact during fluvial transport, we performed a tumbling experiment with rotary tumblers filled with tap water and siliciclastic sediment of different grain size fractions (very fine to medium sand, 63–500 µm; fine gravel, 2–8 mm).

Sandblasting (2) was used to simulate the mechanical effects of aeolian sediment transport. This type of alteration is encountered in fossil or modern vertebrate remains exposed to weathering in arid or desert systems. In such settings, vertebrates remains decompose and disarticulate on the sediment surface and face intermittent sandblasting prior to burial. It is not well known if, and how exactly, this “sandblasting” affects the dental surface. Therefore, we performed a sandblasting experiment with loess and fine sand to assess how different grain size fractions of siliciclastic mineral particles alter dental microwear textures. Loess is a widespread silt-sized aeolian sediment that is often deposited during glacial periods (Wright, 2001). The quartz fine sand used in this study is at the upper end of the typical sediment fraction which can be carried by suspension in dust storms (Squires, 2001).

Finally, we performed an acid etching experiment (3). This type of chemical alteration was chosen because it frequently occurs in small mammals and other vertebrates, which are preyed on by raptorial birds, which then regurgitate bones and teeth in pellets. Small vertebrates are common fossils found in coprolites (*e.g.*, owl pellets) and they could potentially be affected by the strong stomach acid of the predator. Carnivorous and necrophagous birds, like grey falcon, the bald eagle, vultures and different owl species, have stomach pH-values of ≈ 1.0–1.8 (Beasley et al., 2015), while carnivorous mammals have higher pH values, depending on the species (*e.g.*, cat: 3.6, dog: 4.5; Houston & Cooper, 1975; Beasley et al., 2015). Faunivorous sharks (Papastamatiou, Purkis & Holland, 2007) and reptiles, such as crocodiles and snakes, have low stomach pH-values of around 2, at least during digestion (*e.g.*, *Crocodylus niloticus*, Pooley & Gans, 1976; *Python molurus*, Secor, 2003). Thus, for the acid-etching experiment, we immersed different vertebrate teeth in specific concentrations of hydrochloric acid matching the chemical (*i.e.,* pH) conditions of typical predator stomach environments.

For the first time, such extensive alteration experiments of dental surface texture with teeth from different modern taxa, two large mammals, one small mammal, one fish, and one reptile species were performed to cover three key mechanical and chemical post-mortem alteration processes. However, it has to be noted that experiments were not designed to reproduce natural alteration processes faithfully. Instead, key variables (sediment contact during fluvial transport, sediment contact during aeolian transport, acid-etching during digestion) were considered isolated from other effects in order to reduce the complexity of natural post-mortem alteration processes. This allows us to clearly identify the effect of the considered variable. Moreover, the simplified process was also exaggerated compared to naturally occurring intensities, to ensure production of post-mortem wear. We intend to address the following two research questions in this study:

1. Are post-mortem dental wear features resulting from natural processes visually recognisable and do experimentally produced post-mortem wear features resemble them?
2. Are surface texture parameters that are commonly used for DMTA able to detect artificially produced post-mortem alteration?

For this purpose, we applied scale-sensitive fractal analysis (SSFA, *e.g.*, Ungar et al., 2003; Ungar et al., 2012; Scott et al., 2006; Scott, 2012) and 3D surface texture analysis (3DST, *e.g.*, Schulz, Calandra & Kaiser, 2010; Schulz, Calandra & Kaiser, 2013; Schulz et al., 2013; Purnell & Darras, 2016; Kubo et al., 2017; Purnell et al., 2017; Winkler et al., 2019), two different DMTA techniques that evaluate 3D representations of the enamel surface at submicron resolution. For addressing the first research question, we measured the experimentally altered dental surface before and after each experiment and compared the resulting surfaces with those of fossil and extant specimens that potentially underwent comparable natural alteration processes. For addressing the second research question, we applied pairwise comparison of the experimentally altered dental surface before and after the experiment and used principal component analysis (PCA) for evaluation of textural changes.

## Material and methods

### Experimental design

Generally, the experimental designs follow from practicality and availability of equipment and specimens, but are also deliberately simplified to deal with a reduced number of variables and thus enable recognition of simple cause and effect relationships. However, it has to be noted that they represent more extreme but also simplified approximations of natural conditions, and can therefore not be immediately compared to naturally occurring post-mortem wear features. How well the simplified approach reflects naturally occurring alteration is one question addressed in this study. The experiments as presented in this study are the first ones of this extent, approaching different post-mortem alteration scenarios (*e.g.*, fluvial transport, aeolian sediment transport and acid etching due to stomach acid), under different conditions and for several taxa with diverse dental morphologies. Several variables influence post-mortem alteration, such as grain size fraction of colliding particles during fluvial transport and make its effects highly variable (Gümrükçü & Pante, 2018). This hinders the understanding of the underlying processes and the recognition and interpretation of observed wear marks on dental surfaces. The simplified experiments with partially extreme conditions were chosen to ensure distinct post-mortem alteration on the dental surface. Furthermore, the experimental designs allow replicability and the best-possible control of the influencing variables, and therefore comparison between different experimental setups and species.

**Table 1:** Experiment, treatment, species and number of teeth in total of this study.

Experiment	Treatment	Species	No of teeth (total)
Tumbling	Very fine sand	*Equus* sp., *Capreolus capreolus*, *Otomys* sp., *Crocodilus niloticus*, *Carcharhinus plumbeus*	60
	Fine sand		
	Medium sand		
	Fine gravel		
	Natural	*Hippotherium primigenium*	6
Sandblasting	Fine sand	*Mammuthus* sp.	1 (3 slices)
	Loess	*Mammuthus* sp.	1 (3 slices)
	Natural	Antelope indet.	4
Acid etching	Avian predator	*Otomys* sp	3
	Crocodile	*Crocodilus niloticus*	3
	Shark	*Carcharhinus plumbeus*	3
	Barn owl pellet	*Otomys irroratus*	8
	Live-trapped	*Otomys* sp.	8

### Tumbling experiment

The tumbling experiment was designed to simulate the basics of fluvial transport, *i.e.,* contact between river sediment of different grain size fractions and teeth. To ensure a constant movement of sediment and tooth specimens, commercial hobby rotary tumblers TYP TRO 2 A (Otto Eigner e.K., Industriebedarf & Hobbyschleifmaschinen, Idar-Oberstein, Germany) were employed at University Mainz. Similar to Böhm et al. (2019), two different siliciclastic sediments, a quartz-dominated (∼60%) sand and a fine to medium grained gravel, were sieved into four different grain size fractions for tumbling (Tables 1 and S1): very fine sand (51–168 µm), fine sand (112–292 µm) and medium sand (221–513 µm), and fine to medium-grained gravel (2–8 mm). Experimental tumbling barrels were filled with a mixture of 300 g of sediment and 500 g of tap water, tumbling machines run at a constant speed of 45 rpm. The high mixing ratio of sediment and water was chosen to ensure a constant contact between the sediment and tooth and a constant suspension of sediment in the water. This, together with the constant speed of the rotary tumblers is necessary to ensure similar experimental conditions for all grain size fractions and species during the whole experimental duration. The dataset includes dental surface measurements of modern cheek teeth from three different large and small mammals (zebra (*Equus* sp.), roe deer (*Capreolus capreolus*) and African vlei rats (*Otomys* sp.)), one archosaur (Nile crocodile, *Crocodilus niloticus*) and a cartilaginous fish (sandbar shark, *Carcharhinus plumbeus*, Table S2). Three teeth per species and grain size fraction were used for the experiment, resulting in a total 12 teeth per species, and a total of 60 tooth specimens from 5 species (Table S2). Each tumbling experiment was performed with only one tooth per barrel to avoid tooth-tooth contact. Each tooth was tumbled for 336 h (= 2 weeks) and surface texture scans were measured on the same, pre-experimentally marked area of the tooth enamel surface both before and after the experiment. The duration of 336 h of tumbling was chosen based on previous experiments (Böhm et al., 2019), to ensure post-mortem wear feature production. No transport distance was calculated, since rotary tumblers would produce abrasion of the dental surface more rapid than natural fluvial setting, making a direct comparison between artificial and natural transport distances inaccurate (Shipman & Rose, 1988; Gümrükçü & Pante, 2018). It has previously been observed that even 10 km of fluvial transport in a tropical montane system reduced the mass of volcaniclastic pebbles to two thirds of their original mass and resulted in an exponential decline in pebble diameter (Miller et al., 2014). A continuous transport over 336 h represents a very extreme and in natural settings unlikely case of constant movement in a fluvial system and therefore the upper end of possible taphonomic alterations due to fluvial transport. This experimental duration was chosen to maximise abrasion, produce identifiable grain size specific post-mortem wear features during the experiment and to assess post-mortem surface modifications.

Enamel surface scans were obtained directly on the original dental surface, except for, one specimen of roe deer (tx right M3, Table S2), which was moulded using high-resolution silicone-Vinylpolysiloxane precision impression material Provil novo Light regular set EN ISO 4823, type 3, light (Heraeus Kulzer GmbH, Dormagen, Germany). The occlusal surface of this specimen was to oblique to be measured directly on the original dental surface. Scans on original and moulded dental surfaces are considered to have similar quality and contain the same information on wear features (Mihlbachler, Foy & Beatty, 2019). Sample preparation of mammalian teeth is described in detail in Böhm et al. (2019). Data of roe deer, zebra, and *Otomys* teeth tumbled in the three sand-sediment grain size fractions have originally been published in Böhm et al. (2019) and are included as comparison to non-mammalian vertebrates. Four different wear facets of left and right upper premolar and molar teeth were analysed for the large mammals (roe deer and zebra), while enamel lamella L1 and L2 were analysed for the *Otomys* teeth (Table S2, compare Böhm et al., 2019). For sharks, the dental surfaces of the lingual side were analysed (compare Weber et al., 2021a) and on the buccal side for the crocodile teeth. For both crocodiles and sharks, measurements were taken from positions close to the apex and towards the tooth centre. To ensure measurement of the same enamel surface areas after the tumbling experiment, mammalian teeth were marked with cutmarks on enamel bands while crocodile and shark teeth were marked with a small drill hole on the centre of the dental surface. The dental surface was aligned to the reference cutmark/drill hole and measured at the same distance as in the previous scans. Four non-overlapping scans were taken of all experimentally altered teeth (for details see Table S2).

In comparison to this experimentally altered set of dental surfaces, we also analysed cheek teeth of the fossil horse *Hippotherium primigenium* from the Dinotheriensande, Eppelsheim, Germany (Table 1). The Dinotheriensande are Late Miocene (∼10.5 Ma) fluviatile deposits of the ancient Rhine River (Steininger et al., 1996; Andrews & Bernor, 1999). Mammalian fossils in this locality are almost entirely restricted to a siliciclastic, sand- and gravel-dominated horizon close to the base of the sedimentary sequence (Tobien, 1983). The similarity in grain size spectrum of these deposits containing fluvially transported isolated teeth makes them suitable for comparison with our experimentally altered large mammal teeth.

### Sandblasting experiment

The sandblasting experiment was designed to simulate post-mortem alteration due to aeolian sediment transport, using a sandblasting machine (basic quattro IS; Renfert, Hilzingen, Germany) to blast the mineral dust vertically onto the sample from a distance of 30 cm and with a pressure of 3.5 bar for 7–14 s. These experimental conditions were chosen for practical reasons, 3.5 bar is the lowest pressure setting on the sandblaster and a seven-second interval is the shortest human reaction time we could set for turning the sandblaster on and off. The pressure of 3.5 bar represents an approximate wind speed of 100 m/s when the particles hit the dental surface, which is about 3 to 4 times higher than gusts of wind in very strong naturally occurring dust-storms (*e.g.*, up to 30 m/s measured in Chinese dust-storms, Gengsheng, Honglang & Wanquan, 2001). Two different natural sediments were used: Late Pleistocene airborne loess (Schwalbenberg outcrop; Remagen, Germany) as a very common sediment deposited by aeolian transport and Oligocene fluvial fine sand (Feinsand 12c; Dörentrup Quarzwerke, Germany) as a comparison for the larger, sand-sized grain size fraction (Table 1). The silt-sized loess was sieved through a 300 µm mesh and had a mean grain size of 36.87 µm (range: 6.3 to 200 µm, data from laser diffraction size analysis, Table S1), with quartz (37.3 wt.-%), calcite (15.7 wt.-%), illite and mica (13.8 wt.-%), plagioclase (9.8 wt.-%), and dolomite (5.4 wt.-%) as main mineral phases. The fine sand 12c had a mean grain size of 166.01 µm (range: 63 to 630 µm, data from laser diffraction size analysis, Table S1) and was composed of nearly pure quartz (99.5 wt.-%). As sample specimen a single fossil tooth of *Mammuthus* sp. from Pleistocene Rhine River deposits was cut several times parallel to the occlusal surface to produce several slices comprised of multiple enamel and dentine bands. The slices were polished using silicon carbide (SiC) of different grain size fractions down to 6.5 µm, to produce large, smooth enamel surfaces analogous to “blank pages”, where new taphonomic wear features could easily be identified. Four non-overlapping scans were measured on three different enamel bands before and after sandblasting with each of the two sediment types. The experimental duration for sandblasting with the fine sand was 7 s, while sandblasting with loess was prolonged to 14 s after no alteration was observed when initially blasting for 7 s.

For comparison to the artificially produced post-mortem features, naturally “sandblasted” (*i.e.,* eroded by windblown sand) fossil antelope teeth from the Late Miocene Toros-Menalla fossiliferous area in Chad, approximate 500 km NE of the present-day Lake Chad, were analysed (Tables 1 and S2). The fossil-bearing siliciclastic sediments consist of clay and sandstone (Lebatard et al., 2008) with a sediment grain size comparable to that of the fine sand used in our experiment. Most present-day wind-speeds for dust-storms in the Chad are 2 to 6 m/s (Cowie, Knippertz & Marsham, 2014), and thus below our experimental conditions. All specimens were measured on the original dental material.

### Acid etching experiment

The acid etching experiment was designed to simulate acid etching from stomach acid during digestion, using hydrochloric acid to achieve predator stomach specific pH-values, and warming the solution to match temperature conditions experienced in the gastro-intestinal tract of different predators (Tables 1, 2 and S2). A mixing ratio of 10 ml acid to 1 g dental material was applied, and the acid was renewed after 24 h to ensure persistence of the target low pH. The pH-value was kept constant during the whole experimental period. Teeth of *Otomys* sp., *Crocodilus niloticus* and *Carcharhinus plumbeus* were *in-vitro* exposed to acid etching after being tumbled for 336 h in the fine sand fraction (112–292 µm). The samples were chosen for practical reasons, as they were available for destructive experiments, and there were visually few signs of alteration after tumbling, which was also confirmed as we found no significant statistical alteration of the post-tumbling surface compared to the pre-tumbling surface. The measurement positions of the enamel surfaces were similar to the ones from the tumbling experiment. *Otomys* teeth were kept in trace-metal grade 0.1 mol/L hydrochloric acid of pH ∼1.3 for 12 h at a temperature of 45 °C to simulate the stomach conditions of a barn owl prior to the regurgitation of bone and tooth material in the form of pellets (Grémillet & Plös, 1994; Beasley et al., 2015). Crocodile teeth were kept under crocodile stomach conditions, while shark teeth were kept under simulated shark stomach conditions, to simulate stomach conditions of a carnivorous reptile and predatory fish (Table 2). These large predators frequently replace their teeth, and potentially swallow some of them. Fossil shark and crocodile teeth may therefore have passed through their respective stomachs. For both crocodile and shark teeth, we used 0.05 mol/L hydrochloric acid (pH ∼2) at 25 °C for 48 and 72 h to simulate passage times through the gastrointestinal tract of each respective taxon (Pooley & Gans, 1976; Hutton, 1987; Papastamatiou, Purkis & Holland, 2007, Table 2).

**Table 2:** Taxon, number of teeth, pH-value, temperature, and etching interval for the stomach acid alteration experiment. [1] Beasley et al., 2015; [2] Papastamatiou, Purkis & Holland, 2007; [3] Pooley & Gans, 1976; [4] Grémillet & Plös, 1994; [5] Hutton, 1987.

Taxon	Number of teeth	pH value	Temperature [°C]	Etching interval [hrs]
*Otomys* sp.	3	1.3 ^[1]^	42 ^[4]^	12 ^[4]^
*Carcharhinus plumbeus*	3	2 ^[2]^	25 ^[2]^	48 ^[2]^
*Crocodilus niloticus*	3	2 ^[3]^	25 ^[5]^	72 ^[3]^
*Otomys* sp.	8			Barn owl pellet
*Otomys irroratus*	8			None (live-trapped)

After the experiment, all teeth were thoroughly rinsed with Milli-Q water (18.2 M Ω) and dried at room temperature. Two different sets of modern *Otomys* teeth from the Cradle of Humankind World Heritage Site, South Africa (collected in 2017) were used as natural reference samples for comparison to those from the acid etching experiment. The first sample set (*Otomys* sp.) of teeth was extracted from barn owl pellets and served as a direct comparison to the African vlei rat teeth from the etching experiment (Tables 1 and S2). The second sample set of teeth (*Otomys irroratus*) was extracted from skulls of live-trapped animals and provided the natural baseline for non-altered, ingesta-related dental surface textures from the same geographic setting and species. For all specimens DMTA were performed on the original dental material.

### Dental microwear texture analysis

**Figure 1:**
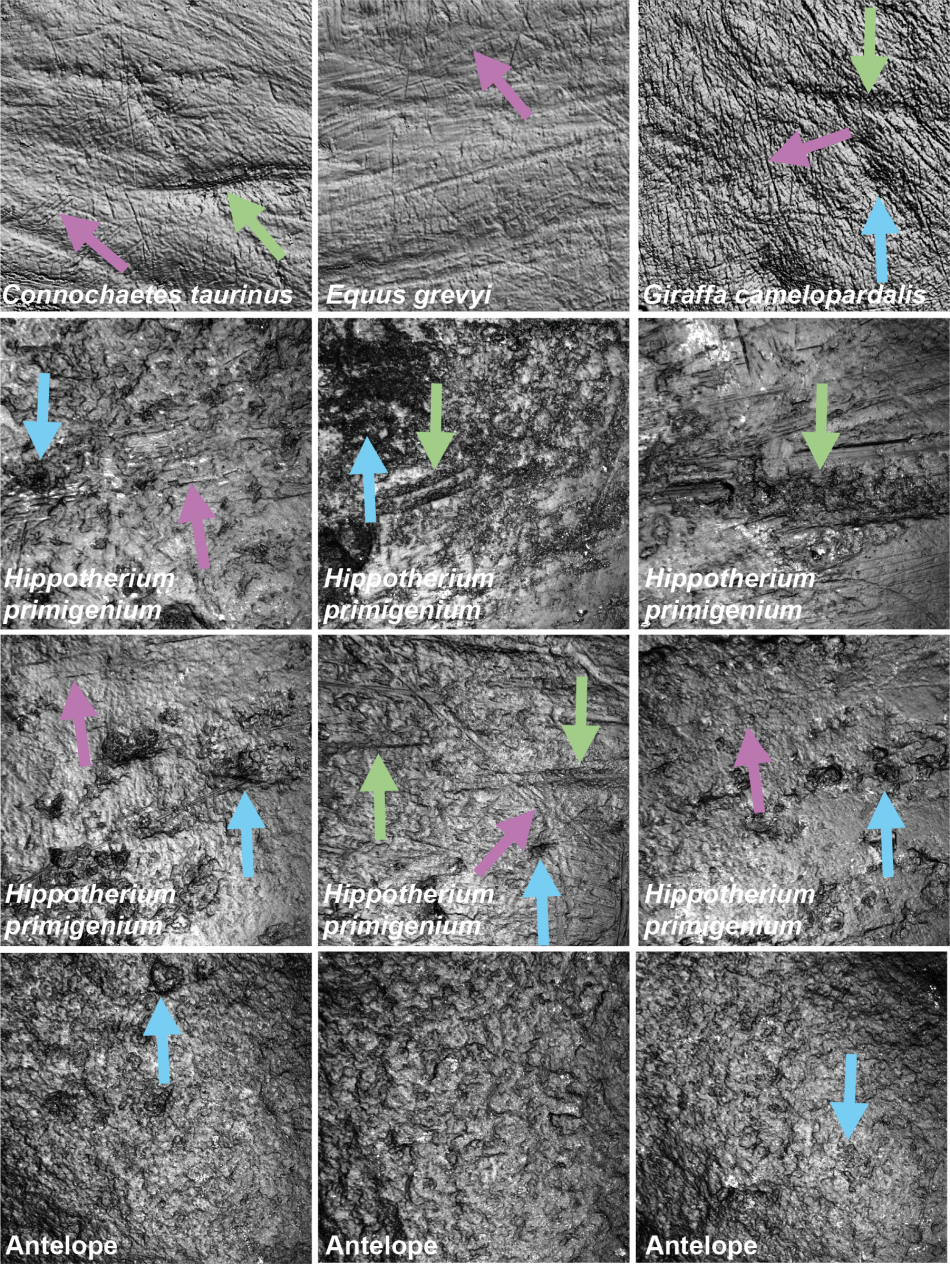
Exemplary photosimulations of ingesta-related and post-mortem enamel wear features (all surface scans 160 × 160 µm) of modern *Connochaetes taurinus*, *Giraffa camelopardalis*, *Equus grevyi* (Schulz, Calandra & Kaiser, 2013) as well as Miocene fossil *Hippotherium primigenium* and antelope teeth (this study). Note, *Hippotherium* teeth are from fluvial deposited gravel-sand whereas the antelope teeth were naturally sandblasted. Violet arrows point to scratches on the dental surface; green arrows indicate furrows and blue arrows dales, on both ante- and potentially post-mortem dental wear surfaces.

All samples were cleaned prior to moulding or scanning using water to remove adherent dirt, ethanol, and acetone to remove lipids and varnishes or glue from the dental surface. Surfaces were scanned using the high-resolution disc-scanning confocal 3D-surface measuring system µsurf Custom (NanoFocus AG, Oberhausen, Germany) with a blue LED (470 nm) and high-speed progressive-scan digital camera (984  × 984 pixel). Four non overlapping square areas of 160  × 160 µm were scanned using a ×100 long distance lens (numerical aperture 0.8) with a resolution in *x*, *y* = 0.16 µm, and *z* = 0.06 µm on each sample. Measurements with an oblique orientation too significant for measurement were rejected (displacement range *δ*z > 60 µm). Following Böhm et al. (2019) for the experimental settings, a rotation along *x*-, *y*- and *z*-axis of the teeth between individual measurements of 5–10° was accepted. We used 3D (enamel) surface texture analysis (see Schulz, Calandra & Kaiser, 2010, Schulz, Calandra & Kaiser, 2013; Schulz et al., 2013; Calandra et al., 2012) and scale-sensitive fractal analysis (SSFA) with parameters after Ungar et al. (2003) and Scott et al. (2006), implemented in MountainsMap Premium v. 7.4.8676. We used three customized templates in MountainsMap Premium v.7.4.8676 software to generate an S-L surface (noise, waviness, and form removed; for more details see Schulz, Calandra & Kaiser, 2013). A detailed description of the templates including the pre-processing procedures are given in the electronical supplements. Subsequently a set of 46 surface texture parameters were quantified on the S-L surface using the following analyses: (1) the ISO 25178, (2) motif, (3) furrow, (4) direction, (5) isotropy, and (6) ISO 12871 (flatness) analysis (Table S3). In this work, it is focussed on the expression of ante- and post-mortem wear features, such as hills and dales. Dales are shallow or deep, roundish “holes” in the dental surface (Fig. 1), with a lowest point (pit); while hills have a highest point (peak); both are defined in the software MountainsMap Premium v.7.4.8676 by watershed algorithms setting its limits along the course and ridge line including the saddle points. Ante-mortem dales are generally short, shallow, and multiple a combination of multiple dales result in an open motif (furrow-like), that is orientated in the same direction, and might be produced during the chewing process. Contrary, post-mortem open motifs are often longer, deeper, and crosscut ante-mortem wear features (Fig. 1). All scans are accessible *via* figshare.

### Statistics

The open-source software R v.4.0.0 (R. Development Core Team, 2020) with the packages xlsx (Dragulescu, 2014), rJava (Urbanek, 2016), doBy (Højsgaard & Halekoh, 2016), R.utils (Bengtsson, 2016), factoextra (Kassambara & Mundt, 2017), plyr (Wickham, 2011), ggplot2 (Wickham, 2016), Rcpp (Eddelbuettel & Francois 2011, Eddelbuettel, 2013), rela (Chajewski, 2009) and WRS version 0.12.1 (Wilcox & Schönbrodt, 2010) were used for data processing and all statistical analyses. A median for each DMT parameter was calculated from four non-overlapping scans. From this median, a mean and standard deviation (1SD) was calculated for all experimental datasets for t_0_ (before each experiment as a baseline) and t_i_ (after each experiment) prior to analysis (Table S4–S6). In general, we employed pair-wise comparisons for the following datasets:

1. Pairwise comparison for dependent data of pre- and post-tumbling surfaces in all four grain size fractions, five species in the tumbling experiment (Table S7).
2. Pairwise comparison for dependent data of pre- and post-sandblasted surfaces in the two grain size fractions used in the sandblasting experiment (Table S7).
3. Pairwise comparison for dependent data of pre- and post-etching surfaces in all three species in the acid etching experiment (Table S7).
4. Pairwise comparison of independent data from *Hippotherium primigenium* and all tumbled species (Table S8), and from trapped animals (*Otomys irroratus*), *Otomys* sp. extracted from owl pellets and experimentally altered *Otomys* teeth before and after acid etching (Tables S2 and S9).

For pair-wise comparison of dependent data the Wilcoxon signed rank test with continuity correction was employed (Wilcoxon, 1945). This test indicates whether the dental surfaces after the experiment differ significantly from the dental surface before the experiment. Pairwise comparison of independent data was subjected to the statistical test routine after Calandra et al. (2012) and Schulz, Calandra & Kaiser (2013a). We applied the robust heteroscedastic Welch-Yuen omnibus test (Welch, 1938; Yuen, 1974) and a heteroscedastic pairwise comparison test (“Lincon test”, analogous to Dunnett’s T3 test (Dunnett, 1980)), according to the procedure of Wilcox (Wilcox, 2003; Wilcox, 2005; Wilcox, 2012). We performed a data trimming of 15% to remove outliers and compensate for non-normality. Finally, we applied the robust heteroscedastic rank-based test after Cliff (Cliff, 1996; Schulz, Calandra & Kaiser, 2013). For principal component analyses (PCA) a set of 12 parameters was selected according to the following reasons: best separating parameters (mammals: Schulz, Calandra & Kaiser, 2013; reptiles: Winkler et al., 2019), independent parameters from different parameter categories (Table S3) and no missing values. Before applying PCAs, we use the Kaiser-Meyer-Olkin test for sampling adequacy (*p*-value > 0.5) and Bartlett’s test of sphericity (<0.05) to validate the application of a principal component analysis (PCA) is justified on our data. PCAs were conducted using the built-in function prcomp(). Principal components (PCs) with standard deviation over 1.0 are discussed in the following. Factor loadings were cut at a value of 0.4 (Budaev, 2010), describing the best separating parameters in the different PCs (Table S10).

## Results

### Tumbling experiment

#### Very fine sand (51–168 *μ*m)

**Figure 2:**
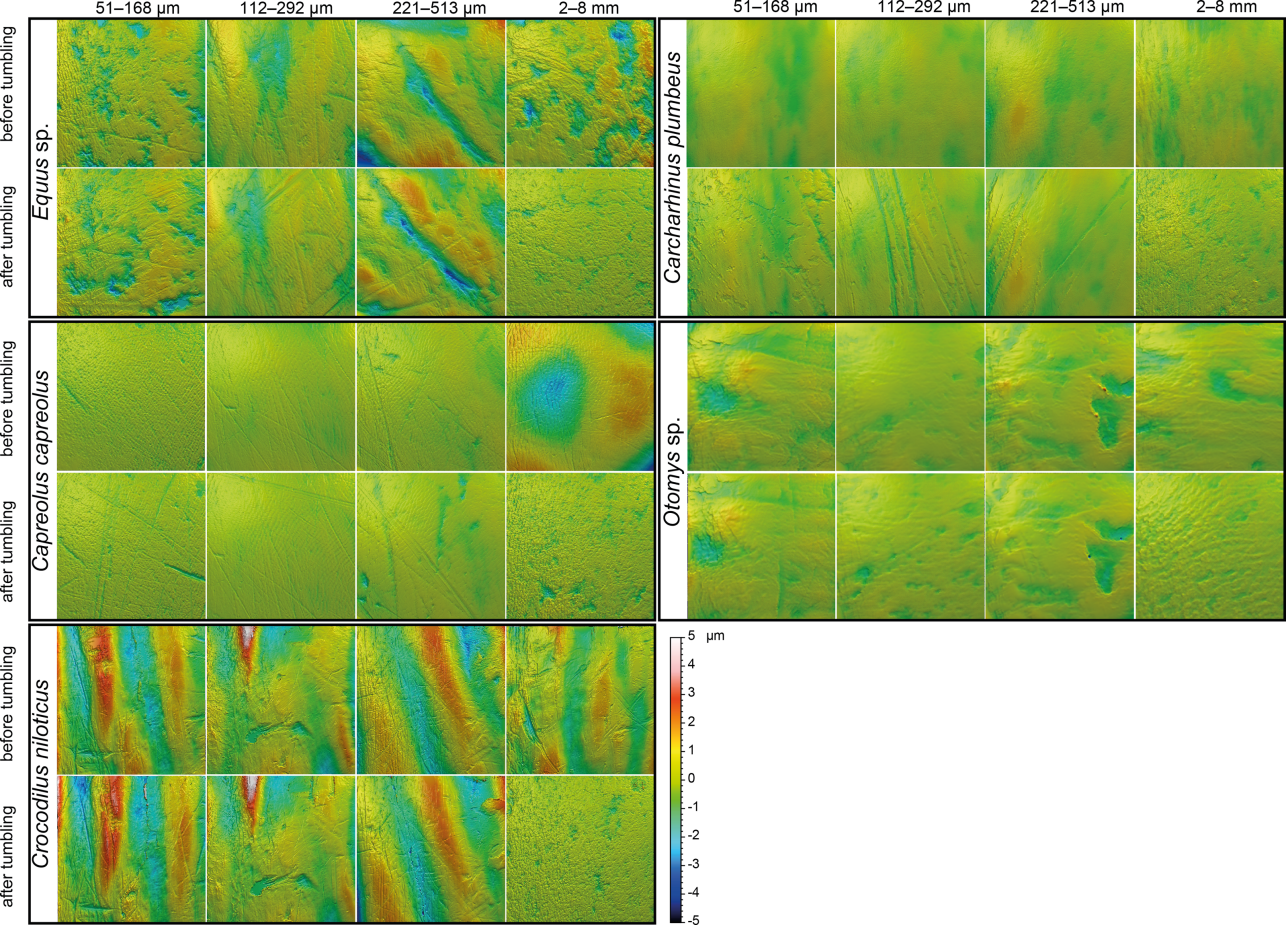
Meshed axiomatic 3D models of enamel surfaces. Enamel surfaces of the 3D models (160 × 160 µm if not specified otherwise) represent experimentally altered teeth before tumbling and after tumbling for 336 h with four different grain size fractions of siliciclastic sediment: very fine sand (51–168 µm), fine sand (112–292 µm), medium sand (221–513 µm) and fine to medium gravel (2–8 mm). Zebra: *Equus* sp.; roe deer: *Capreolus capreolus*; crocodile: *Crocodilus niloticus*, and shark: *Carcharhinus plumbeus*; African vlei rat: *Otomys* sp., scan size 60 × 60 µm.

**Figure 3:**
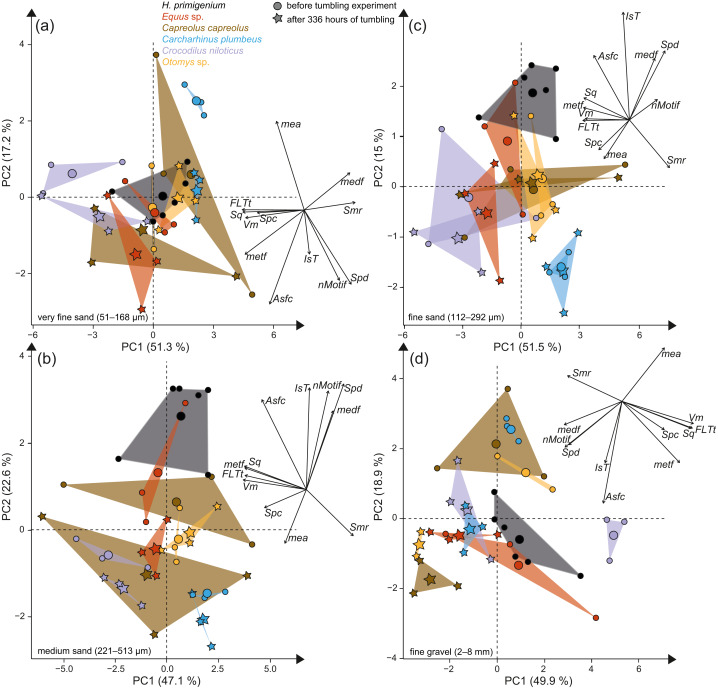
Principal component analysis (PCA) of surface texture parameters from each category (Table S3). Large symbols in the PCA plot mark the mean area of the different groups; for details about the taxa see Table S2. (A) very fine sand (51–168 µm, (B) medium sand (221–513 µm), (C) fine sand (112–292 µm) and (D) fine to medium gravel (2–8 mm).

The first column of Fig. 2 shows exemplary 3D enamel surface models of experimentally altered dental surfaces before (first row) and after the experiment (second row) for each of the five used species. Experimental alteration of teeth with very fine sand led to a higher number of new wear features, such as new small and large dales, which crosscut ante-mortem wear features and show no preferred direction (larger anisotropy values of parameters, except for *Otomys*, Figs. S1A and S2A). The post-tumbling dental surface of *Carcharhinus plumbeus* shows additionally a large number of new dales (Fig. 2). This coincided with an increase in overall complexity of the surface (larger *Sdr*, *Asfc* values, except for *Otomys*). For the large mammal teeth (*Equus* and *Capreolus*, Table S4), an increase in height and volume parameters was found. The *p*-values from the Wilcoxon signed rank test indicate significant differences between the complete dental surface before and after the tumbling experiment for *Capreolus capreolus*, *Crocodilus niloticus*, *Carcharhinus plumbeus* and *Otomys* sp., while *Equus* sp. did not show significant surface modifications (Table S7). The PCA displays separation of dental surface before and after tumbling for all five different species along PC1–PC3 (Figs. 3A and 3SA), whereby the isotropy (*IsT*), complexity (*Asfc*), area (*mea*), density (*Spd*) and plateau size (*Smr*) show the highest factor loadings and therefore explain more variance in the PCA (Table S10).

#### Fine sand (112–292 *μ*m)

Tumbling the teeth of the five different species with the fine sand resulted in only minor modifications on the dental surface (Fig. 2, second column of each species). Small new dales were added to the dental surface of the tumbled specimens, which is best visible on the dental surface of *Carcharhinus plumbeus* (Fig. 2). Quantitative analyses of the surface texture parameter values of teeth tumbled in this grain size fraction indicated only slight changes in the direction of the wear features (texture direction) with changing complexity and fewer peaks and dales (Table S4). Only *Capreolus capreolus* showed significant changes of the whole dental surface before and after the experiment, signified by the Wilcoxon rank test (Table S7). The PCA therefore shows no separation of dental surfaces before and after tumbling, except for *Equus* sp. (PC1 and PC2, Figs. 3C and S3C) and for *Charcharhinus plumbeus* (PC3, Fig. S3C).

#### Medium sand (221–513 *μ*m)

Similar to the tumbling with the fine sand, tumbling with medium sand resulted mostly in slight surface modifications, only small new post-mortem dales appear, which cross-cut ante-mortem features, *e.g.*, *Equus* sp. (Fig. 2, column 3). Additionally, a slight polishing effect occurs, which is indicated by a decreasing complexity in the biplot of *Asfc vs. epLsar* (Fig. S1B), and a decreasing density of the open dale motifs (furrow-like) in the biplot of *metf vs. medf* (Fig. S2B). However, the whole dental surface was only significantly modified during the experiment for *Otomys* sp. and *Crocodilus niloticus* according to the Wilcoxon rank test (Table S7). The PCA shows a separation in PC2 and PC3 (Figs. 3B and S3B) for all specimens expect *Otomys* sp. The parameters with the highest factor loadings and therefore the explanation for more variance in the PCA are material volume (*Vm*), peak curvature and peak density (*Spc* and *Spd*), number of motifs (*nMotif*), mean area (*mea*) as well as the isotropy (*IsT*).

#### Fine to medium-grained gravel (2–8 mm)

Tumbling with 2-8 mm-sized gravel led to major changes in surface texture patterns and resulted in a complete overwriting of the dental surface. A high number of small dales are produced (Fig. 2, fourth column of each species) and very similar texture features on dental surfaces between different species become apparent after the experiment, which indicates significant post-mortem abrasion. However, contrary to the sand grain size fractions, no visible post-mortem dales were added during tumbling with the fine gravel. Both the complexity (*Asfc*, Fig. S1D) and the isotropy of the dental surface increased in all specimens (Table S4). Parameters describing the height, plateau size, slope, and volume showed different trends in the teeth of different species (Table S4). The specimens tumbled with gravel had a high mean density of furrows with a low depth, plotting as a data cloud in the biplot of *medf*—mean density of furrows *vs. metf*—mean depth of furrows parameter space (Fig. S2D). The surface texture parameter values for all five species experimentally altered using gravel, displayed a huge overlap with the parameter space of the fluvial transported fossil *Hippotherium primigenium* teeth from the Dinotheriensande (Fig. S2D). The Wilcoxon signed rank test indicated significant changes of the whole dental surface before and after the experiment only for the *Equus* sp., *Crocodilus niloticus* and *Otomys* sp. (Table S7). A PCA revealed a strong separation of dental surface before and after the tumbling for *Capreolus capreolus*, *Crocodilus niloticus*, *Carcharhinus plumbeus* and *Otomys* sp. (Figs. 3D, 2D and S3D). The parameter with the highest factor loadings and therefore the explanation for more variance in the PCA are the peak curvature (*Spc*), complexity (*Asfc*) and isotropy (*IsT*).

### *Hippotherium primigenium* teeth from the Dinotheriensande

Figure 1 shows photosimulations of *Hippotherium primigenium* enamel surface scan from the Dinotheriensande Eppelsheim, Germany, a fluvial deposit with siliciclastic, sand- and gravel-dominated dominated sediments (Tobien, 1983). The surface scans show clear differences in wear features to ingesta-related dental surfaces of modern grazing and browsing species (Fig. 1), *i.e., Connochaetes taurinus*, *Giraffa camelopardalis*, *Equus grevyi* (Schulz, Calandra & Kaiser, 2013). *Hippotherium primigenium* teeth show a large number of dales, together with a large number of long furrow-like features (Figs. 3 and S2), however, a very low number of narrow dales (scratch-like features). Dales are aligned in the same direction, crosscutting and overwriting ante-mortem wear features (Fig. 1). Generally, the dental surfaces appear to be very rough, compared to the ingesta-related dental surfaces of modern herbivorous ungulates (Fig. 1). However, *Hippotherium primigenium* shows only a few significant differences to *Equus* sp. before the experiment, while the number of significant differences increases after the experiment for the fine and medium sand fraction and the fine gravel fraction (Table S8). *Capreolus capreolus* differs significantly in one to three parameters before the experiment in the sand fractions, and in 20 parameters in the fine-gravel fraction. After the tumbling experiment, the number of significant differences decreases for the sand fractions slightly and increases for the fine to medium gravel fraction. Statistical comparisons between *Otomys* sp. and *Hippotherium primigenium* were only possible for the sand fractions and reveal an increase of significant differences for the very fine and medium sand and a decrease for the fine sand fraction. *Crocodilus niloticus* and *Carcharhinus plumbeus* show both a high number of significantly different DMT parameters compared to *Hippotherium primigenium* before the experiment, and a strong decrease in number of significant differences after the tumbling with the very fine sand and the fine gravel fraction. After tumbling with the fine and medium sand fractions, the number of significant differences is increasing for *Crocodilus niloticus* and decreasing (fine sand) or remains stable (medium sand) for *Carcharhinus plumbeus.*

**Figure 4:**
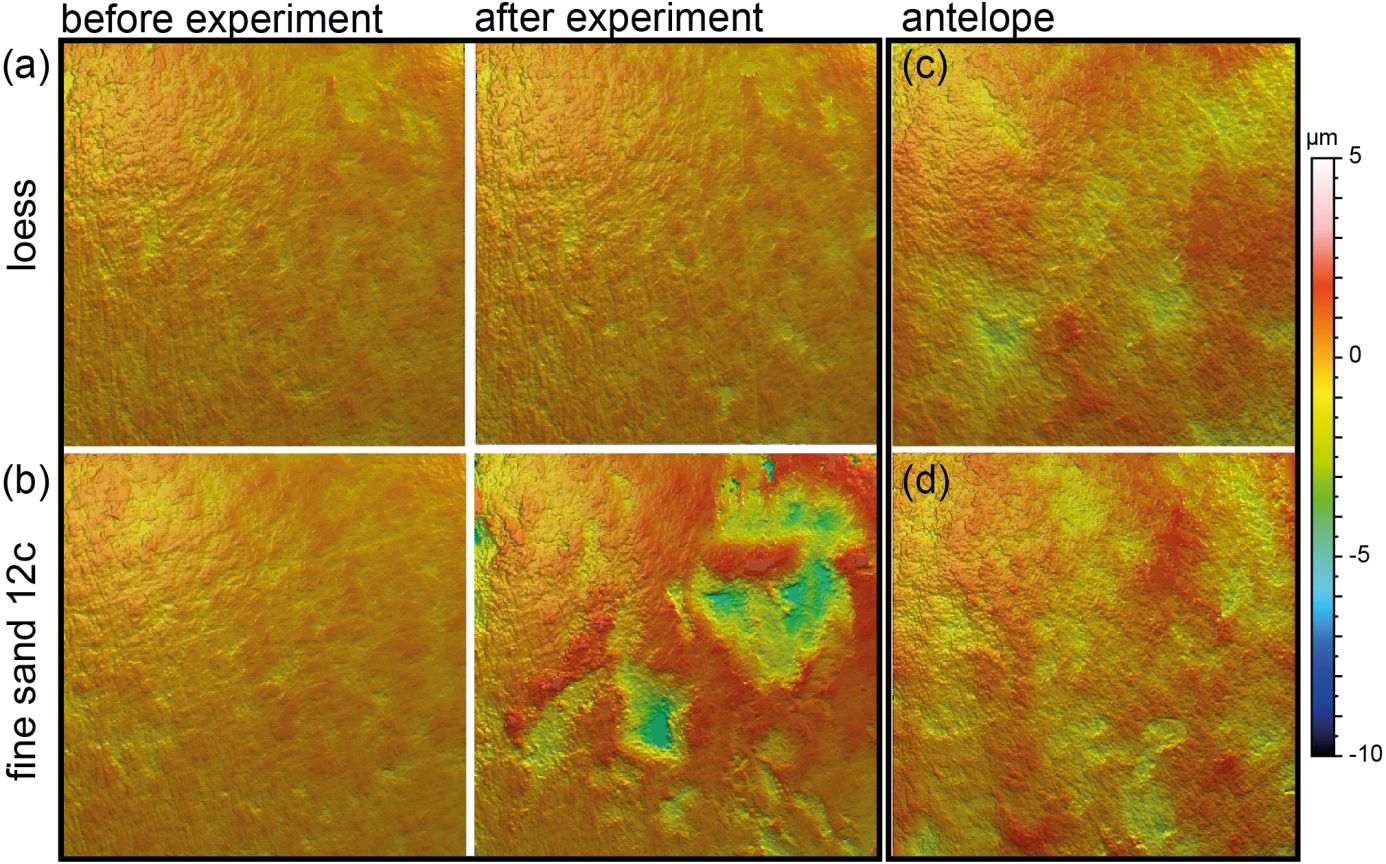
Meshed axiomatic 3D models of enamel surfaces (160 x 160 µm) of the experimentally sandblasted teeth of the same area of tooth specimens before (left) and after (right) alteration. (A) *Mammuthus* sp. before and after being sandblasted with loess for seven seconds; (B) *Mammuthus* sp. before and after being sandblasted with fine sand 12c for seven seconds; (C) and (D) Miocene antelope tooth from Chad (Lake Chad) as natural example of a fossil tooth that was exposed to windblown mineral dust (comparable to fine sand 12c in particle size) in comparison to tooth surfaces from the sandblasting experiment.

### Sandblasting experiment

**Figure 5:**
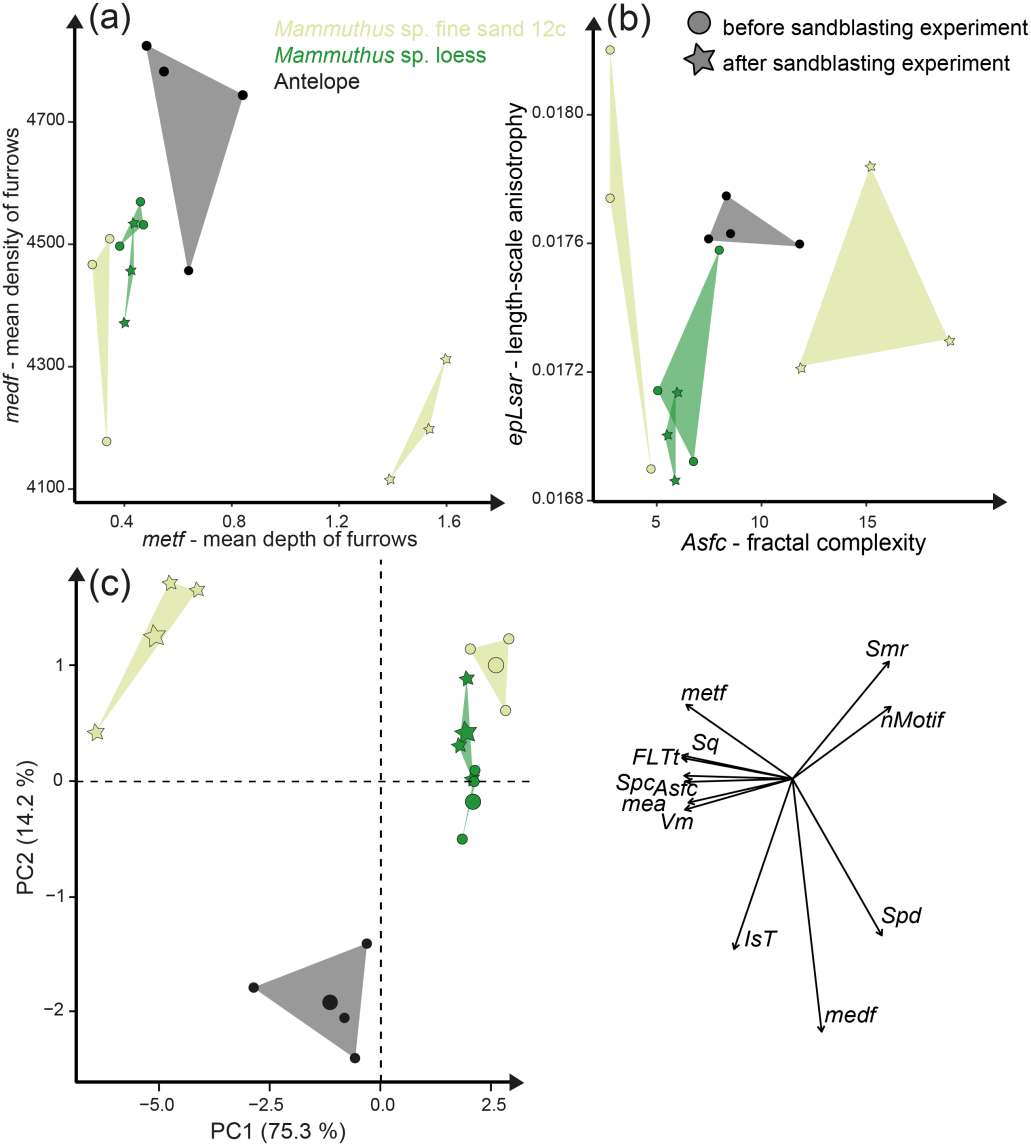
Results of the sandblasting experiment. Dental microwear texture parameter values (A) *metf* (µm) *vs. medf* (cm/cm^2^) and (B) *Asfc vs. epLsar* for the experimentally altered polished mammoth enamel surfaces before (circles) and after (stars) the sandblasting experiment. (C) Principal component analysis (PCA) of surface texture parameters from each category (Table S3). Large symbols in PCA plot mark the mean area of the different sample sets.

Sandblasting the *Mammuthus* sp. polished surface with loess did not change the surface texture pattern; at least not after 14 s of blasting time (Figs. 4A and 5; Tables S5 and S7). The impact of the fine sand 12c however, was very prominent, and resulted in a significant difference between dental surface before and after sandblasting derived from the Wilcoxon Signed-Rank test, with numerous new large dales comparable to impact craters, formed on the surface of the section (Figs. 4B and 5, Table S7). The biplot of *medf*—mean density of furrows *vs. metf*—mean depth of furrows displayed strong separation of surface parameter values before and after the experiment with strongly increasing depth of the furrows (Fig. 5A). The biplot *Asfc vs. epLsar* also showed this strong separation for sandblasting with fine sand, with strongly increasing fractal complexity (*Asfc*, Fig. 5B). PCA revealed strong separation of dental surface before and after the sandblasting (Fig. 5C), with material volume (*Vm*) and depth of the furrows (*metf*) having the highest factor loadings.

Additionally, we compared the experimentally sandblasted mammoth dental surfaces with potential post-mortem features of naturally “sandblasted” fossil antelope teeth from the Late Miocene Toros-Menalla, Chad. Generally, the antelope teeth show no sign of ingesta-related ante-mortem wear compared to ante-mortem dental surface of other modern large ungulates (Fig. 1). The dental surface of the fossil specimens shows a rough dental surface with small to large dales, however, not as large as the enamel surface experimentally altered with the fine sand 12c. The biplots of *medf vs. metf* show high *medf* values, while *metf* is similar to the mammoth dental surface before and after sandblasting with loess (Fig. 5A). The PCA reveals a strong separation of antelope and the experimentally altered mammoth teeth (Fig. 5C).

### Acid etching experiment

**Figure 6:**
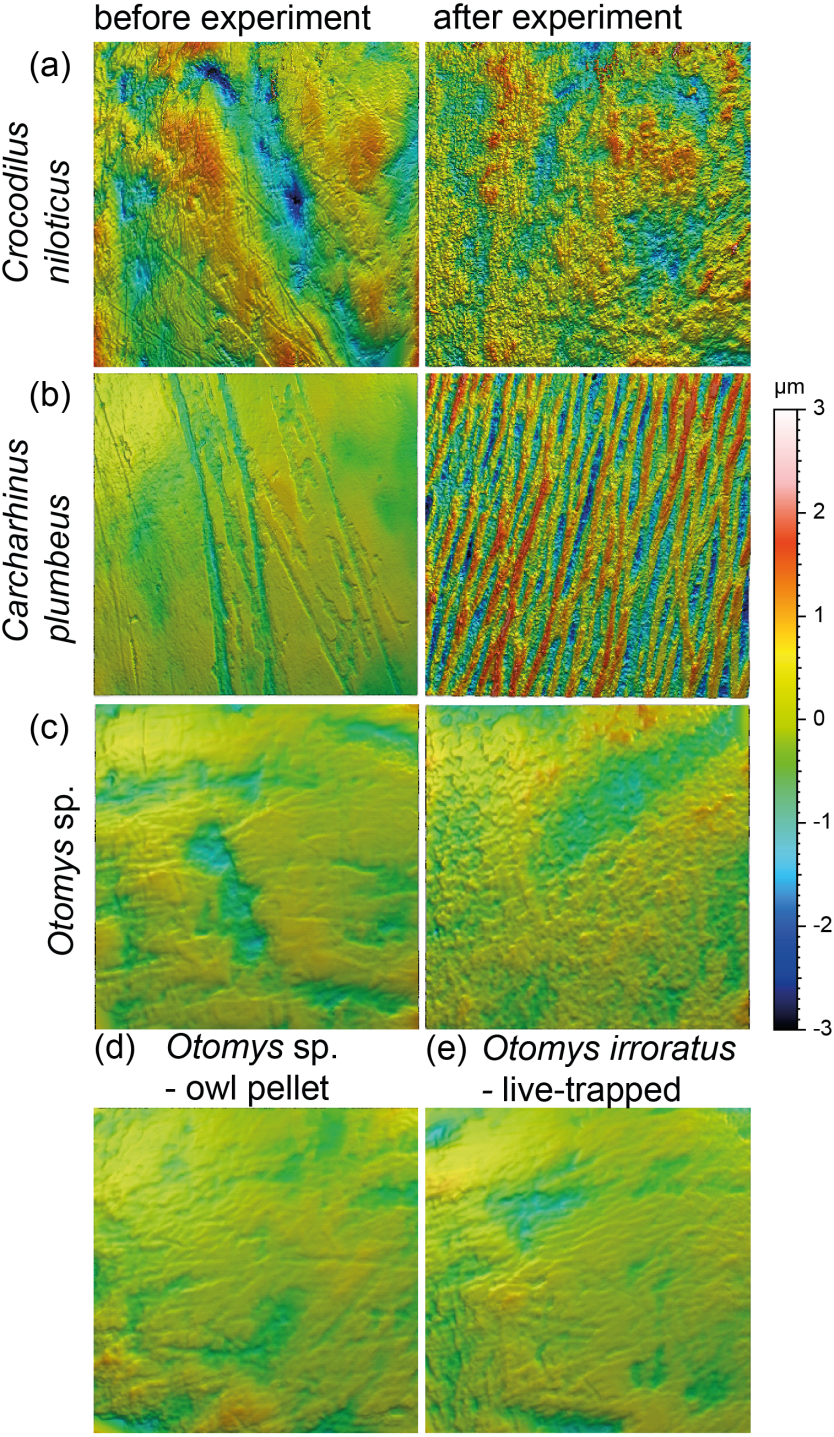
Meshed axiomatic 3D models of enamel surfaces (160 × 160 µm) of dental specimens before (left) and after (right) the acid etching experiments. Scan size is 160 × 160 µm except for all *Otomys* sp. for which scan size is 60 × 60 µm. (A) *Crocodilus niloticus* before and after experiment at pH 2 for 72 h; (B) *Carcharhinus plumbeus* before and after experiment at pH 2 for 48 h; (C) *Otomys* sp., before and after experiment at pH 1.3 for 12 h; (D) *Otomys* sp. from modern owl pellets as natural comparison to the acid etching experiment; (E) *Otomys irroratus* live-trapped as unaltered comparison to the naturally altered *Otomys* sp.

**Figure 7:**
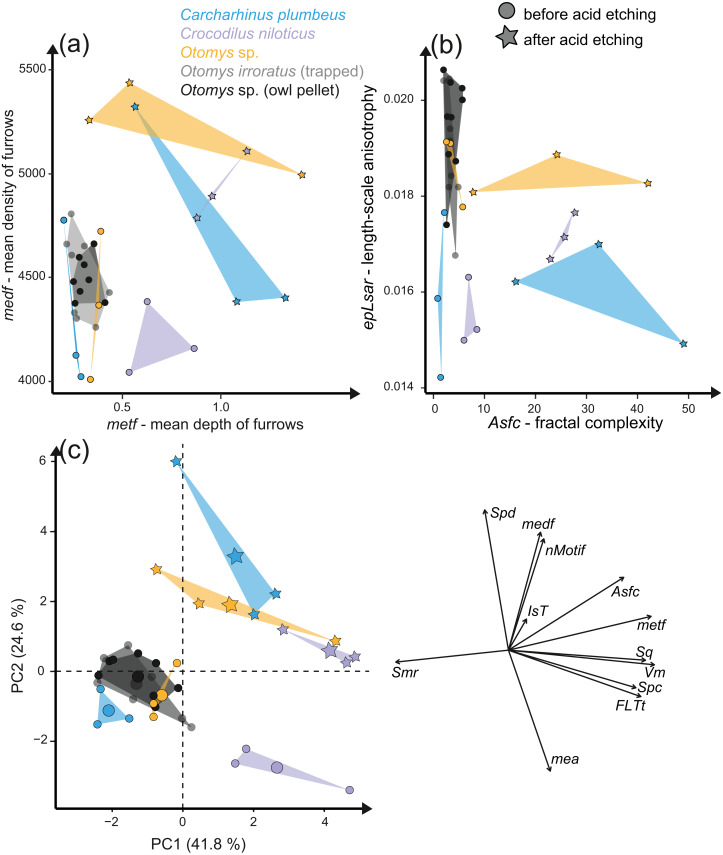
Results of the acid etching experiment. Dental microwear texture parameter values (A) *metf* (µm) *vs. medf* (cm/cm^2^) and (B) *Asfc vs. epLsar* for the experimentally altered teeth before (circles) and after (stars) the acid etching experiment. (C) Principal component analysis (PCA) of surface texture parameters from each category (Table S3). Large symbols in PCA plots mark the mean for each taxon/etching interval.

The acid etching experiment was performed with three different species, *Otomys* sp., *Crocodilus niloticus* and *Carcharhinus plumbeus* at different pH-values and temperatures. Overall, it is evident that the outermost layer of enamel (consisting of hydroxylapatite) and enameloid (consisting of fluorapatite) is significantly affected by etching during the experiment (Fig. 6) and thus the original dental surface is completely overwritten. The dental surface is very rough without clear signs of specific post-mortem features, such as narrow furrow-like features. The greatest alteration is visible for *Carcharhinus plumbeus*, where the height of the surface increases strongly (Fig. 6). The Wilcoxon Signed-Rank test indicates significant textural surface modification after the acid etching for all three species (Table S7). The biplot *medf vs. metf* as well as *Asfc vs. epLsar* displayed a strong separation in all three species with increasing density and depth of furrows, as well as increasing complexity of the dental surface after the etching (Figs. 7A and 7B). PCA revealed a strong separation (Figs. 7C and S5) with the highest factor loading in the parameters density of the peaks (*Spd*), number of motifs (*nMotif*), depth of furrows (*medf*) and mean area (*mea*) parameters (Table S10).

Additionally, we compared two sets of different natural African vlei rat tooth samples—one live-trapped and the other digested and extracted from barn owl pellets—with each other and with the experimentally altered *Otomys* teeth. The two natural samples are barely distinguishable from each other according to all surface texture parameters (Figs. 6D and 6E, Fig. 7, Table S10). Even though Dunnett‘s T3 test displays significant differences between the two natural samples sets as well as the experimentally altered *Otomys* teeth, the overlap of the parameter values is very large and therefore none of the differences were confirmed by Cliff’s method (Table S10). The biplot *medf vs. metf* and *Asfc vs. epLsar*, as well as the PCA displayed strong separation of the two natural *Otomys* sample sets from the experimentally altered *Otomys* samples after the acid etching (Fig. 7, Fig. S5).

## Discussion

### Fluvial transport in a sediment suspension simulated via a tumbling experiment

Tumbling of mammal, reptile and fish teeth shows that mechanical alteration of dental surfaces as characterised by dental microwear texture analysis seems to be more dependent on the sediment particle size than on the type of enamel(-oid) microstructure, tooth morphology or size (Fig. 2). The dental surfaces of different species are strongly affected by sediment abrasion, especially by the fine gravel fraction. Very fine sand and gravel have the strongest alteration effects on abrasive texture patterns over the tumbling two-week time interval. The surfaces altered by the fine gravel fraction look quite similar across species (Fig. 2) and they share a similar parameter space in the biplot *metf vs. medf* (Fig. S2), as well as in the PCA (Fig. 3). Ante-mortem wear features are completely overwritten with post-mortem wear features, *i.e.,* small and large dales. However, no post-mortem furrow-like features are added. The alteration effect of the very fine sand grain size fraction is characterized by the formation of numerous long narrow and deep furrows crosscutting ante-mortem wear features and each other. Additionally, the formation of post-mortem dales, connected to narrow post-mortem furrows, is detectable, however, fewer than during the experiment with the fine gravel fraction (Fig. 2). In contrast, tumbling with the fine and medium sand had a slight polishing effect resulting in only a few post-mortem scratch-like features. The effects of the tumbling experiment with these grain size fractions are mostly undetectable by statistical analyses (Table S7). In a direct comparison of the pre-tumbling with the post-tumbling surfaces, a significant separation was found in the very fine sand and the fine gravel fraction. The separation was visible for the biplots as well as in the PCA; however, still with a substantial overlap. Volume and overall height parameters seem to be least affected. This is not surprising, since parameters of these groups describe the whole surface and are therefore less affected by addition of singular wear features. Parameters from these groups are among the best for distinguishing between different feeding strategies (*e.g.*, browser-grazer, Schulz, Calandra & Kaiser, 2013; herbivorous, carnivorous, frugivorous and molluscivorous lepidosaurs, Winkler et al., 2019) and less affected by simulated diagenetic alteration (fluvial transport, Böhm et al., 2019). However, diagenetic alteration caused by fluvial transport can be critical for dietary reconstructions using DMTA and requires careful sample selection prior to scanning to exclude taphonomical altered teeth or surface areas. Dental specimens from fossil assemblages found in river sediments with small gravel fractions might show very rough dental surfaces with a low number of dales and furrows, which look very similar between species. Specimens from a more sandy environment might express more scratch-like features or slightly abraded dental surfaces, depending on the grain size fraction. The comparison to the fossil equid teeth *Hippotherium primigenium* from the Dinotheriensande, which were fluvially transported to a certain extent and deposited in sand-gravel sized siliciclastic sediments (Tobien, 1983), reveals similarities between the natural and the experimental surface alteration features from the fine gravel fraction (Fig. 3D). Generally, our tumbling experiments revealed that fossil teeth transported in fluviatile settings with gravel or sand, could be altered and that ante-mortem wear features might be erased or at least modified mechanically during transport due to post-mortem alteration. Therefore, a proper training in recognising various taphonomic alterations helps to serve this purpose. We recommend that such training should include experience in recognizing various taphonomic alterations, which should ideally be calibrated to a database of surface textures characteristic of poorly preserved enamel surfaces as described in Weber et al. (2021b). Here, we provide a first set of post-mortem wear feature examples produced by artificial fluvial transport with specific grain size fractions. Similar post-mortem wear features could be expected on potentially fluvial transported fossil specimens.

The experimental setup as presented in this study has advantages as well as limitations. The advantage is the guaranteed constant transport of sediment and teeth of different species in the same time interval, and therefore for the same distance. This ensures a replicability and comparability across different species, independent of tooth weight or size (Shipman & Rose, 1988). This would not be possible for experiments in flow channels, for instance. Therefore, for a simplified, replicable, and controllable experimental design, rotary tumblers are the ideal tool (Gümrükçü & Pante, 2018). The disadvantage of the simplified tumbling experiment is that it is not able to simulate the very complex process of fluvial transport in a river system. Fluvial transport is potentially short, depending on different factors, such as size of the fossil and flow rate of the river system, *e.g.*, smaller sediments could be transported over longer distances, in contrast to larger and heavier grains or fossils, where the flow rate must be much faster to transport the material (Harms, Southard & Walker, 1982). The alteration process could be variable and might include movement of sediment around the fossil, reworking or discontinuous draining and flooding processes, leading to not only various post-mortem alterations, but that might also affect the proportion of species in a fossil assemblage (Dodson, 1973; Russel et al., 1996; Läng et al., 2013; Pesquero, Alcala & Fernandez-Jalvo, 2013). The experimental design of this study provides the basic knowledge of the expression of ante-mortem wear features formed by different grain size fraction for a very specific and constant speed and duration of transport. The interaction between different parameters could then be simulated in experimental setups which simulate natural conditions more precisely, *e.g.*, in flow channel experiments.

### Aeolian abrasion simulated via sandblasting experiment

Loess is a typical silt-sized aeolian sediment predominantly deposited during glacial periods in many areas and could potentially have altered post-mortem teeth of the contemporaneous fossil fauna. However, despite the high air pressure and fast speed of sediment particles hitting the dental surface, short duration silt-sized (Ø 36 µm) loess blasting experiments do not lead to any visible alteration and formation of post-mortem wear features or changes of surface texture parameter values (Figs. 4 and 5). Fossil teeth from natural loess deposits are therefore likely usable for diet reconstruction employing DMTA, since natural wind speed are much lower than the one applied in this experiment. In contrast, sandblasting of dental surfaces with larger, sand-sized (Ø 166 µm) quartz grains caused major damage to and abrasion of the enamel surface together with the formation of large post-mortem dales, comparable to impact craters (Fig. 4). This leads to strongly increasing complexity and volume parameters (Fig. 5). Fossil tooth specimens that were naturally or artificially sandblasted using sand-sized sediment grain size fractions seem to be affected by mechanical surface modification and showing comparable post-mortem wear features, *i.e.,* large dales (Fig. 4).

Our sandblasting experiment demonstrated the difficulties of such simplified experimental approaches for testing the impact of aeolian sand on dental surfaces exposed to post-mortem or post-depositional weathering by airborne mineral dust. The air pressure applied in our experiment was very high (*e.g.*, at least 20 times higher than present-day natural wind-speeds in the Chad, Cowie, Knippertz & Marsham, 2014) and constant, leading to excessive alteration of the enamel surface by the quartz fine sand. However, it was also demonstrated, that despite the high air pressures, loess-sized grain size fractions appear not to form post-mortem wear features during natural or artificial aeolian sand transport. Due to our experimental design using only two grain size fractions, *i.e.,* loess (Ø 36 µm) and sand-sized (Ø 166 µm) quartz, it remains unclear which particle size starts to cause the formation of dales on the dental surface, and under which wind speeds.

Additionally, besides the implication of the experimental results on erosion due to aeolian sediment transport, there are implications on the effect of sample preparation using air-abrasive techniques, *i.e.,* sandblasting during preparation of fossils from the embedding sediment/rock. In general, various materials, such as Aluminium oxide, crushed glass, plastic, but also walnut shells or wheat starch, with different hardness, shapes and particle size are used for sample preparation (for detailed information see Graham & Allington-Jones, 2018). The commercial sandblasters used for sample preparation are comparable to the one used in the performed experiment, with similar air-pressure, however, even closer distance to the sample. Therefore, formation of post-mortem wear features, as seen in this study, especially for preparation using larger grain size fractions, seems to be plausible. Air-abrasive techniques should thus be avoided when dealing with dental surfaces for later dietary analyses using DMTA, however, if it is not possible to avoid this, special care needs to be taken to protect the dental surfaces from the blast. Specimens prepared with air-abrasive techniques, especially larger and harder abrasive powders comparable to the quartz finesand (Feinsand 12c) from this study, should be evaluated carefully for potential post-mortem alteration. Suspicious wear features, *i.e.,* large dales, comparable to the features produced using the quartz fine sand (Feinsand 12c), should be expected and therefore avoided.

### Stomach digestion alteration effects simulated via in-vitro acid etching experiments

Acid-etching experiments of mammal, reptile, and fish teeth with diluted HCl alter the original dental surface of enamel (hydroxylapatite: *Otomys* sp., *Crocodilus niloticus*) and enameloid (fluorapatite: *Carcharhinus plumbeus*) by dissolution processes. The ante-mortem wear features are completely overwritten during the experimental duration (Fig. 6). The complexity and the roughness of the dental surface increase strongly and the textures of the dental surfaces are significantly different before and after the etching experiment (Fig. 7, Table S7).

The *in-vitro* stomach acid experiment design performed with isolated teeth directly immersed only in hydrochloric acid do not account for the role of digestive enzymes or buffering effects by the carcasses and therefore resemble more closely the digestive system of crocodiles, where stomach fluids consist almost entirely of hydrochloric acid with almost no enzyme activity (Fisher, 1981). A similar experiment performed by Fernández-Jalvo et al. (2014) showed great similarities between the surfaces of bones and teeth of small mammals immersed exclusively in hydrochloric acid and bones digested by crocodiles. Additionally, they found differences between surfaces treated with hydrochloric acid immersion alone and natural digestion of avian predators as well as mammalian carnivores, which we can confirm with our comparison of experimentally altered *versus* natural *Otomys* samples from owl pellets. We find the surface textures of the experimentally altered teeth to be very different from those of small mammal teeth digested by avian predators. While the ante-mortem wear of experimentally altered *Otomys* samples is completely dissolved and significantly different from both, the dental surface before the experiment and the two sets of non-experimental *Otomys* samples, no significant differences in DMT parameters could be detected between non-digested and digested teeth, *i.e.,* from barn owl-pellets (that were regurgitated after a digestion for at least 10–12 h (Grémillet & Plös, 1994)) and live-trapped specimens, both from the same area in South Africa. For avian predators and mammalian carnivore’s digestion is driven by the combination of stomach fluids and enzymes. Modifications in form of corrosion and decalcification on bones and teeth of small mammals can be reproduced with experimental approaches, but to a lesser degree and at a slower alteration rate than during natural digestion (Fernández-Jalvo et al., 2014). Our results further support the findings of Fernández-Jalvo et al. (2014), that hydrochloric acid erodes the outer tooth enamel layer almost completely, especially the most exposed areas, such as cusps or lamella of molars. The non-existent difference between digested and non-digested teeth of *Otomys* (compare Fig. 6) is in line with findings that in barn owl stomachs, only 1% of ingested molars evidence digestion-related acid-etching and supports the categorization of barn owls in the weakest predator category 1 (*i.e.,* exhibit no or minimal acid digestion, Fernández-Jalvo et al., 2016). We conclude that small mammal teeth extracted from barn owl pellets can be used for diet reconstruction studies using DMTA, however, we did not test if this is also the case for other owl species. It is likely that, at least for birds of prey of predator category 1 (see Fernández-Jalvo et al., 2016), ingesta-related enamel surface textures of prey vertebrates can survive the weak acid attack. Further study of DMTA from small mammal teeth recovered from owl pellets of other species can hopefully confirm our initial results, thus confirming the suitability of such material for dietary reconstruction of small mammals.

## Conclusions

All of our experiments represent simplified and partly exaggerated approximations of post-mortem alteration and do not reproduce the complex, natural conditions teeth will experience during post-mortem taphonomic processes. Nevertheless, both simplified and extreme experimental conditions, can still be very informative in understanding how dental surfaces can be affected by post-mortem wear and alteration processes. To recognise post-mortem features on the dental surface and to assign them to the related taphonomic processes it is necessary to perform experiments with only a very limited number of influencing parameters, such as changes in grain size fraction. Here a first catalogue of potential post-mortem alterations due to taphonomic scenarios is presented.

An important finding of our tumbling experiment is that the effects of tumbling were not as significant as expected, at least not for sand-sized particles. Distinct surface alteration was found in the fine gravel fraction, with a high number of small post-mortem dales that replaced the original diet-related wear features. Very fine to medium sand produced post-mortem scratch-like features and a slight polishing, however, ante-mortem wear features were still identifiable. These grain size fractions frequently occur in natural river sediments, therefore, post-mortem dales might be an issue in fossil specimens from sandy river sediments. Post-mortem scratches and dales produced by tumbling often show a similar orientation and crosscut other features and such specimens should thus be avoided.

Our sandblasting experiment showed that silt-sized aeolian transported mineral particles do not affect the dental surface and only larger, sand-sized quartz particles cause post-mortem abrasion in form of large dales. Depending on the fossil locality and the associated grain size fraction of the aeolian sediment our experiment suggests that sandblasted specimens might be included in DMTA studies. However, special care needs to be taken with specimens altered by aeolian activity with larger grain size fractions. Additionally, fossil tooth specimens prepared using air-abrasive techniques, especially with larger and harder abrasive powders, can suffer from post-mortem wear.

The acid etching experiment revealed a more complex impact of digestion on the dental surface than expected, at least for avian predators. First, the comparison of naturally digested (from barn owl pellets) and un-digested *Otomys* teeth suggest that such small mammal teeth from barn owl pellets can also be used for diet reconstruction using DMTA, at least for samples processed by category 1 avian predators (Fernández-Jalvo et al., 2016). Teeth digested by other carnivores, which have much stronger stomach acids should be carefully evaluated prior to DMTA, since the ante-mortem wear might completely be overwritten.

Our results demonstrate that, at least for a subset of samples from a given fossil assemblage, it is necessary to know which type of post-mortem wear features are expected during which taphonomic process and therefore to be able to identify suspicious looking wear features to evaluate the degree of post-mortem alteration. In this study, we only cover isolated, simplified parameters (grain size fraction, pH-value) of three different post-mortem scenarios (fluvial and aeolian sediment transport, digestion). Still, such simplified experimental approaches can be beneficial. Dental microwear analysis is more automated and less-observer biased than classical 2D microwear analysis, but when it comes to sample selection it easily suffers from subjective bias, and the question whether the features we observe are truly diet-related. Experiments, even though exaggerated or simplified, can create common ground on which researchers can agree on, and identify non-diet related wear features, especially since we expect to recognize post-mortem wear features visually based on our experience, while they are not always significant anomalies in the data. Thus, if identifying post-mortem dental wear objectively through statistical approaches does not work reliably, quality of the dataset might be negatively affected, and we need experimental approaches to create common ground. We highly encourage conducting more elaborated experiments covering other scenarios, such as burial or trampling. Future experiments should then include variations in the influencing parameters of taphonomic processes, such as mixtures of sediment of larger particle size spectra as well as abrasives with different mineralogical compositions. Also, experiments simulating transport in a flow channel instead of tumbling barrels would better to approximate natural fluvial depositional settings. However, since the influencing parameters of taphonomic processes are less controllable during these kinds of experiments, the interpretation might be more complex. Additionally, *in vitro* digestion experiments should be performed with other environmental control (*i.e.,* controlled pH, addition of digestive enzymes) and *in vivo* feeding experiments with actual predators digesting prey and investigation of teeth collected from faeces would complement the dataset.

## Supplemental Information

10.7717/peerj.12635/supp-1 Supplemental Information 1 Supplemental Materials and Figures

10.7717/peerj.12635/supp-2 Supplemental Information 2 Supplemental Tables

10.7717/peerj.12635/supp-3 Supplemental Information 3 Raw data Experiment 1: tumbling experiment; experiment 2: sandblasting experiment; experiment 3: acid etching experiment.

## Additional Information and Declarations

### Data Availability

The following information was supplied regarding data availability: The data are available at figshare: Weber, Katrin; Winkler, Daniela E.; Schulz-Kornas, Ellen; M. Kaiser, Thomas; Tütken, Thomas (2021): Read me. figshare. Dataset. https://doi.org/10.6084/m9.figshare.14974863.v1
Weber, Katrin; Schulz-Kornas, Ellen; Winkler, Daniela E.; Kaiser, Thomas; Tütken, Thomas (2021): Statistics. figshare. Dataset. https://doi.org/10.6084/m9.figshare.14979936.v2
Weber, Katrin; Winkler, Daniela E.; Schulz-Kornas, Ellen; Kaiser, Thomas; Tütken, Thomas (2021): Fossil samples. figshare. Dataset. https://doi.org/10.6084/m9.figshare.14979921.v2
Weber, Katrin; Winkler, Daniela E.; Schulz-Kornas, Ellen; Kaiser, Thomas; Tütken, Thomas (2021): Acid etching experiment. figshare. Dataset. https://doi.org/10.6084/m9.figshare.14979918.v2
Weber, Katrin; Winkler, Daniela E.; Schulz-Kornas, Ellen; Kaiser, Thomas; Tütken, Thomas (2021): Sandblasting experiment. figshare. Dataset. https://doi.org/10.6084/m9.figshare.14979882.v2
Weber, Katrin; Winkler, Daniela E.; Schulz-Kornas, Ellen; Kaiser, Thomas; Tütken, Thomas (2021): Tumbling experiment. figshare. Dataset. https://doi.org/10.6084/m9.figshare.14979849.v1
