# Supplementary material for: Post-mortem enamel surface texture alteration during taphonomic processes—do experimental approaches reflect natural phenomena?
Source: PeerJ. 2022 Jan 14;10:e12635. doi: 10.7717/peerj.12635 (PMC8763041; doi:10.7717/peerj.12635)
Supplement: Supplemental Information 1 [file peerj-10-12635-s001.docx]

**Electronical Supplement**

**Post-mortem enamel surface texture alteration during taphonomic processes ‒ do experimental approaches reflect natural phenomena?**

Weber, Katrin ^1^, Winkler, Daniela E. ^1,2^, Schulz-Kornas, Ellen ^2,3,4^, Kaiser, Thomas M.^2^, Tütken, Thomas^1^

^1^Applied and Analytical Palaeontology, Institute of Geosciences, Johannes Gutenberg University, Mainz, Germany, daniela.winkler@uni-mainz.de, tuetken@uni-mainz.de

^2^Center of Natural History (CeNak), University of Hamburg, Hamburg, Germany, Thomas.Kaiser@uni-hamburg.de

^3^Department of Cariology, Endodontology and Periodontology, University of Leipzig, Leipzig, Germany, Ellen.Schulz-Kornas@medizin.uni-leipzig.de

^4^Department of Human Evolution, Max Planck Institute for Evolutionary Anthropology, Leipzig, Germany

*to whom correspondence should be addressed at katrin.weber@uni-mainz.de

Operators used in MountainsMap template:

*Otomys* template. This template applies to all *Otomys* specimens. The enamel ridge of Otomys are very small, therefore, first a sub-surface of 60 × 60 μm was cut out of the texture model using the extraction operator in MountainsMap. The following MountainsMap template was produced for extracted scans of *Otomys* sp. upper right teeth, scanned on the original tooth. All single teeth with unknown position were treated similarly.

1. Orientation of dental surface with LS-surface using subtraction
2. Noise-cancelling median filter, filter size 5 x 5
3. Gaussian filter, filter size 3 x 3
4. Filling of non-measured points
5. Set the threshold using the material-% (0.5-99.5 %) to reduce height and depth
6. Removal of isolated outliers, outliers at the edge and with a maximum allowed inclination (85°), soft strength of the method, filling of non-measured points on the result-surface
7. Removal of form using a 2^nd^ order polynomial.

Mammal template. This template is for analyses of original upper right premolar and molar teeth of all large mammal species in this study and the polished mammoth teeth. All moulded surfaces were mirrored along the z-axis, to be inverted first. Lower jaw teeth were treated the same way.

1.-6. Similar to *Otomys* template

7. Removal of form using a 2^nd^ order polynomial.

Reptile template. This template is designed for original upper right teeth of crocodile teeth; all teeth were treated the same way.

1.-6. Similar to *Otomys* template

7. Removal of form using a 4^th^ order polynomial.

Shark template. This template is designed for original upper right teeth of sandbar shark teeth; all teeth were treated the same way.

1.-6. Similar to *Otomys* template

7. Removal of form using a 2^nd^ order polynomial.


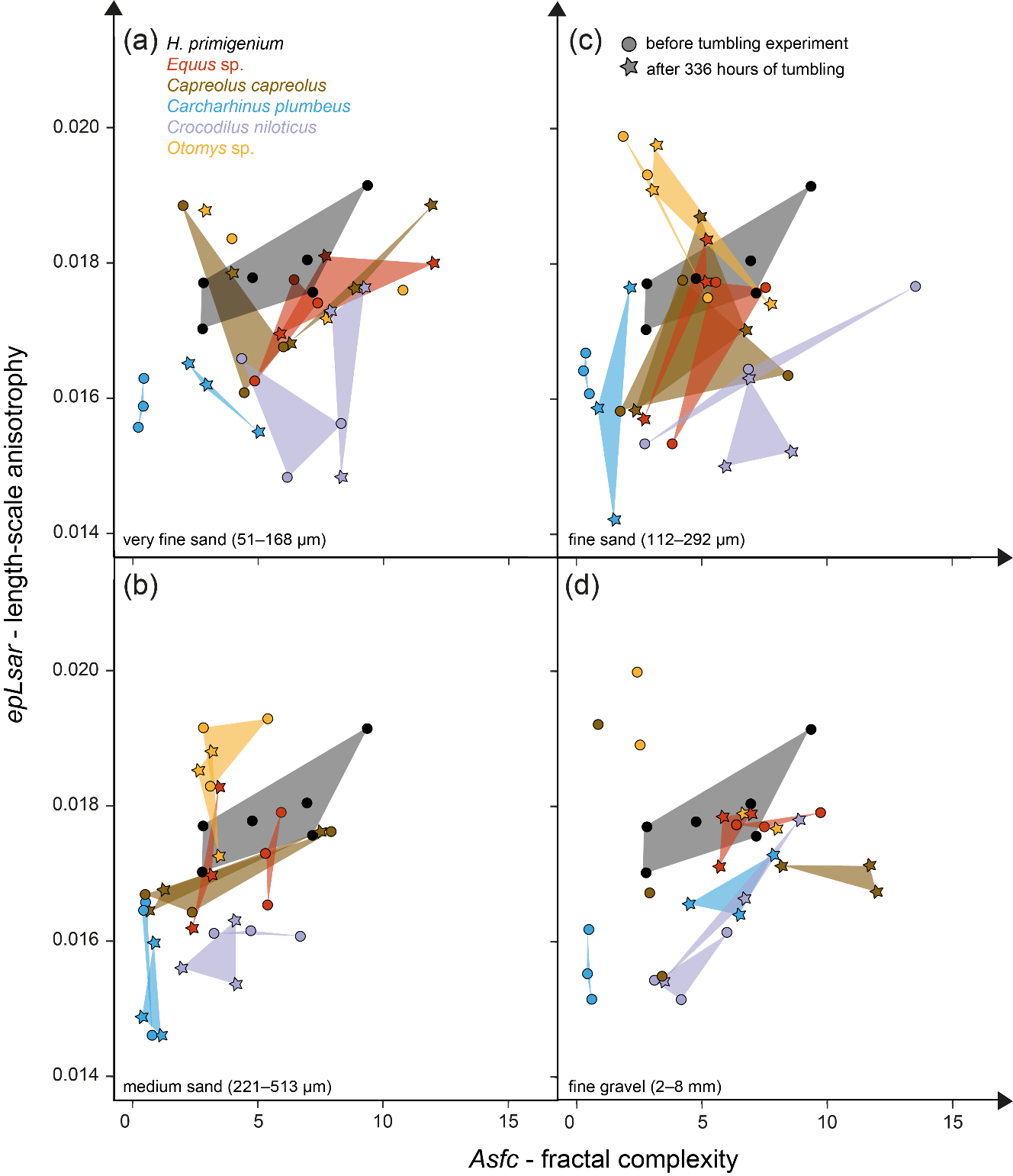


Figure S1: Biplot of the enamel surface texture parameters *Asfc* – fractal complexity versus *epLsar* – length-scale anisotropy for the experimentally altered teeth of different vertebrate species before (circles) and after (stars) the tumbling experiment with (a) very fine sand (51–168 μm), (b) medium sand (221–513 μm), (c) fine sand (112–292 μm) and (d) fine-grained gravel (2–8 mm) as suspension in water.


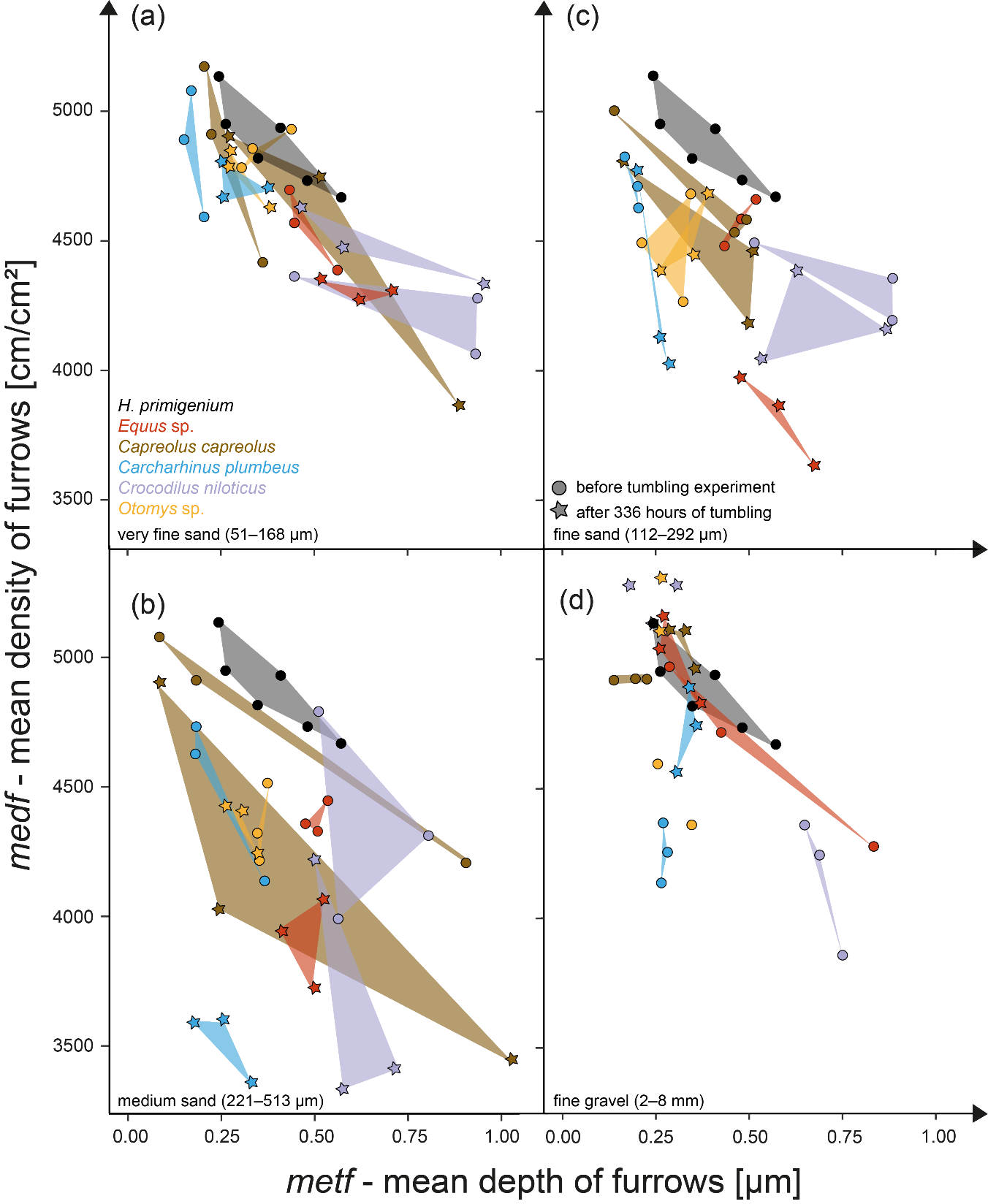


Figure S2: Biplot of the enamel surface texture parameters *metf* (µm) vs *medf* (cm/cm²) for the experimentally altered teeth of different vertebrate species before (circles) and after (stars) the tumbling experiment with (a) very fine sand (51–168 μm), (b) medium sand (221–513 μm), (c) fine sand (112–292 μm) and (d) fine-grained gravel (2–8 mm) as suspension in water.


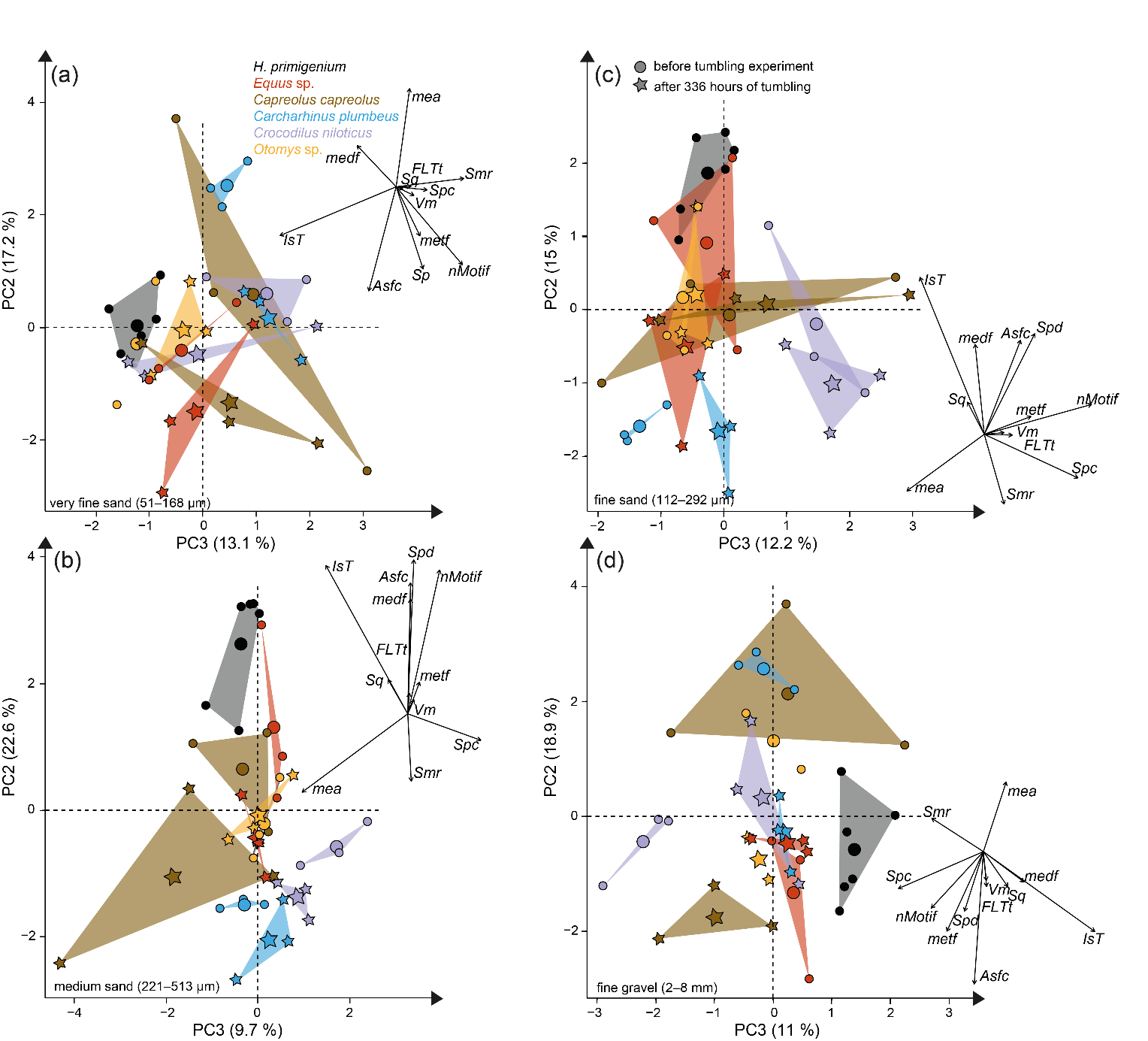


Figure S3: Principal component analysis (PCA) of surface texture parameters from each category (Table S3) for the experimentally altered teeth of different vertebrate species before (circles) and after (stars) the tumbling experiment with (a) very fine sand (51–168 μm), (b) medium sand (221–513 μm), (c) fine sand (112–292 μm) and (d) fine-grained gravel (2–8 mm) as suspension in water. Large symbols in PCA plot mark the mean of the area of the different taxa and tumbling intervals.


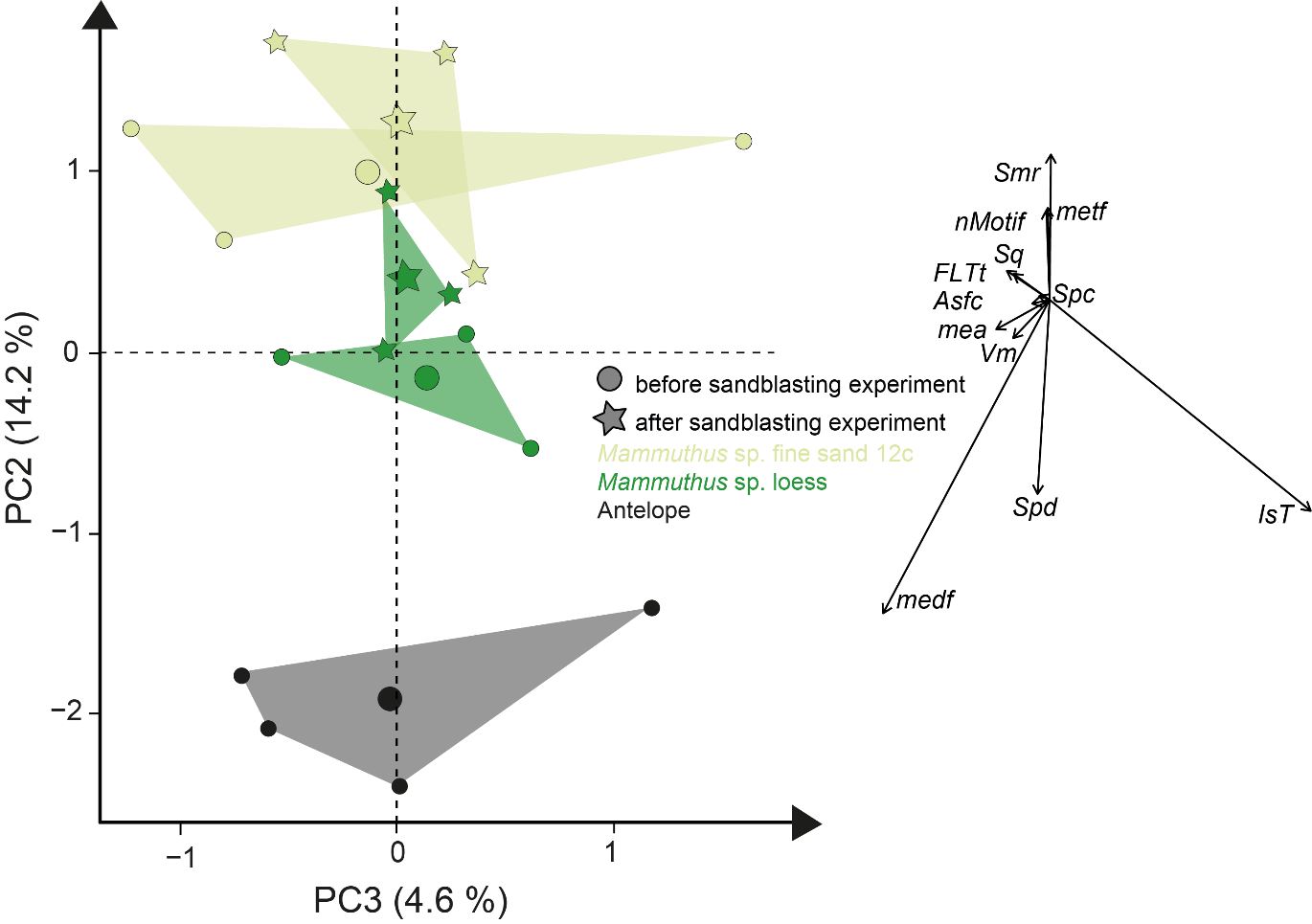


Figure S4: Principal component analysis (PCA) of surface texture parameters from each category (Table S3) for the experimentally altered mammoth teeth before (circles) and after (stars) the sandblasting experiment. Large symbols in PCA plot mark the mean of the area of the taxa and blasting intervals.


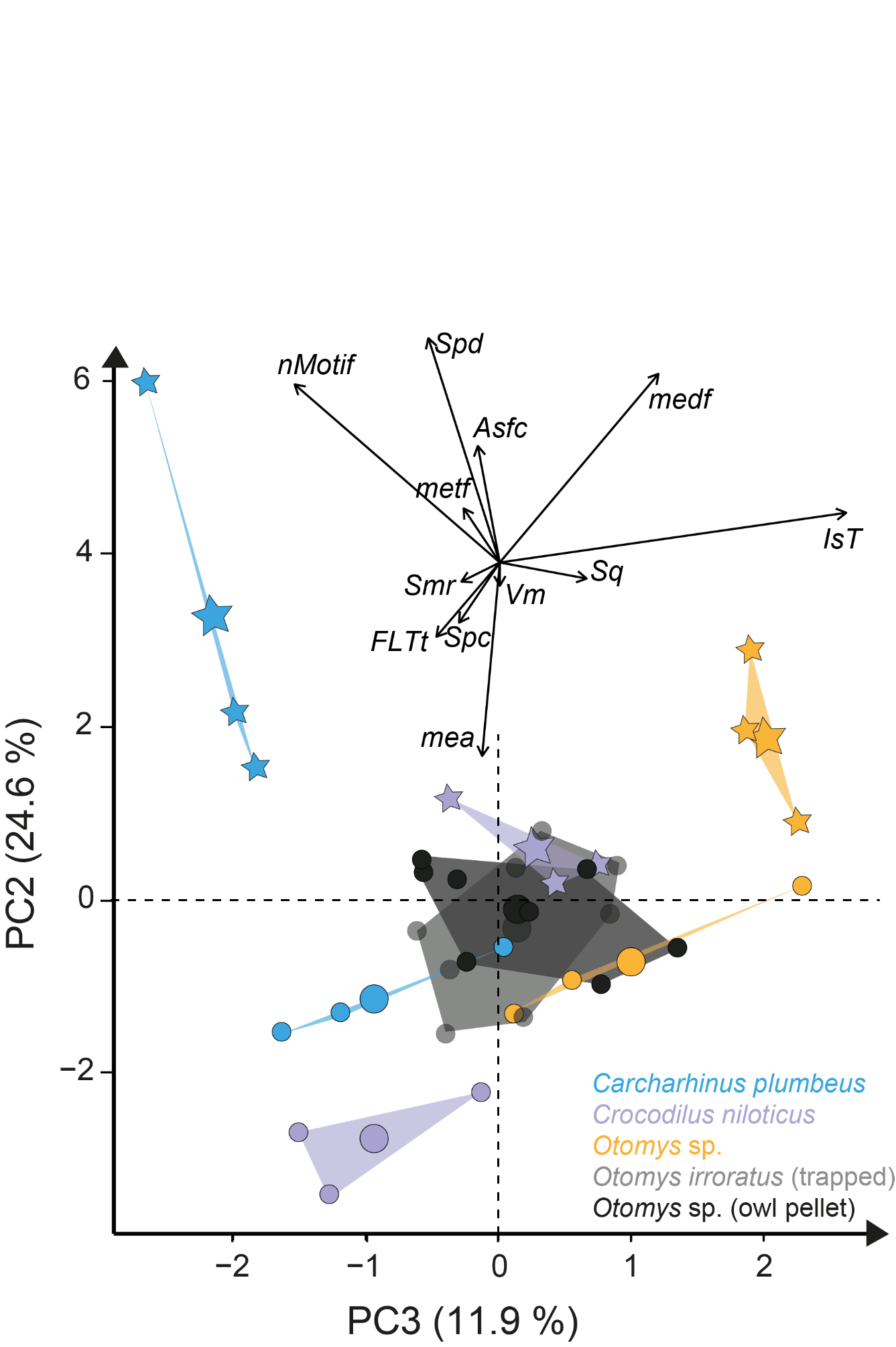
 Figure S5: Principal component analysis (PCA) of surface texture parameters from each category (Table S3) for the experimentally altered mammoth teeth before (circles) and after (stars) the acid etching experiment. Large symbols in PCA plot mark the mean of the area of the taxa and blasting intervals.
